# Boosting the Diversity of a Similarity-Aware Genetic Algorithm Using a Siamese Network for Optimized S-Box Generation

**DOI:** 10.3390/e28040460

**Published:** 2026-04-17

**Authors:** Ishfaq Ahmad Khaja, Musheer Ahmad, Louai A. Maghrabi

**Affiliations:** 1 Department of Computer Engineering, Jamia Millia Islamia, New Delhi 110025, India; ishfaqkhawaja312@gmail.com; 2 Department of Software Engineering, College of Engineering, University of Business and Technology, Jeddah, Saudi Arabia; l.maghrabi@ubt.edu.sa

**Keywords:** genetic algorithm, crossover, diversity, substitution-box, loss function, similarity learning, Siamese network

## Abstract

A difficult NP-hard optimization problem, designing cryptographically robust substitution-boxes (S-boxes) necessitates a careful balancing act between several conflicting properties, such as differential uniformity and nonlinearity. Genetic Algorithms (GAs) have been widely used for this task; however, their performance is often limited by premature convergence and insufficient diversity during crossover operations. This primarily occurs because genetic algorithms commence with limited a priori knowledge. This sort of “blindness” and failure to utilize local knowledge results in diminished performance. In GA, the crossover operations facilitate the dissemination of robust candidates within the population. Conventionally, GA implements crossover for each pair of parents for diversity and a robust solution. However, this is not invariably the situation. To enhance children’s candidacy, parental diversity is quite crucial. This paper proposes a similarity-aware crossover strategy, integrated with a Siamese learning framework, to guide the genetic algorithm for improved S-box optimization with better diversity and faster convergence by utilizing parental local information. The proposed model is similarity-aware to guarantee that the GA improves parental diversity. When the parents exhibit excessive similarity, a “regressive” crossover is opted, which ensures the propagation of a parental couple with sufficient diversity to produce superior offspring. The proposed similarity-aware GA model is applied and evaluated to generate cryptographically robust and optimized S-boxes. To verify the robustness in terms of diversity, the model has been tested using three different loss functions: contrastive loss, KL divergence loss, and the suggested method of combining both loss functions to form a hybrid loss function. The effectiveness of the proposed approach is demonstrated through the generation of high-quality S-boxes with strong cryptographic properties.

## 1. Introduction

Charles Darwin stated the principle of “survival of the fittest” to show how adaptability and diversity drive evolutionary systems. Evolutionary Algorithms (EAs) apply nature-inspired mechanisms to solve complex optimization problems through the concept that extends from biological systems into computational intelligence. The Genetic Algorithm (GA) is one such foundational evolutionary algorithm, as it effectively solves complex problems in engineering, finance, and machine learning domains where traditional methods become ineffective [1]. The Genetic Algorithm (GA) improves candidate solutions through crossover, mutation, and selection operations while maintaining a balance between new and existing solution exploitation in each iteration [1,2,3]. Genetic Algorithms have been extensively applied in systematic design of cryptographic components, specifically Substitution-boxes [3]. The Substitution-box can be described as a nonlinear substitution mapping *S*(*x*): GF(2^n^) → GF(2^m^) (where GF stands for Galois Field) via the Boolean function *f*(*x*) = (*f*_1_*(x*), *f*_2_(*x*),…, *f_m_*(*x*)) [4]. As substitution boxes are the main components responsible for introducing confusion in encrypted data, AES (Advanced Encryption Standard) and other modern symmetric key block ciphers depend on them as their non-linear element [5]. They play a vital role in the defense against the powerful attacks, including linear and differential cryptanalysis. The S-box is the only dynamic component of block ciphers, employed to introduce confusion through a nonlinear mapping of input and output values. The design of a cryptographically robust S-box is considered an NP-hard optimization problem, requiring the simultaneous compliance of multiple properties like high nonlinearity, bijectivity, and low differential uniformity [5]. Consequently, the systematic design of optimal S-boxes remains an active and vital area of research [6].

Even though GAs have proven beneficial, they have certain shortcomings, such as slow convergence rates and getting stuck in local minima. These shortcomings stem from the algorithm’s nature; it often starts with little background knowledge and does not make use of it during the search process [3,7,8]. The crossover operation illustrates this inefficiency most clearly. Standard GAs apply crossover to all parents indiscriminately, assuming that their offspring will always be an improvement over the parents. However, if the parents are too closely related (very similar), the crossover will result in offspring with very little genetic diversity, causing redundant computations and stagnation in the population, resulting in convergence to a local minimum. This loss of parental diversity directly weakens the algorithm’s performance and incapacitates the algorithm in finding the global optimal solution [3]. Among various evolutionary algorithms, we choose the Genetic Algorithm (GA) as the most relevant method since the problem at hand is based on permutations, i.e., the construction of an S-box. We avoid methods such as particle swarm optimization (PSO) or differential evolution (DE), where permutations are not the primary object. The crossover operator, which is fundamental for the genetic algorithm, will also play an essential role in our proposal because we intend to learn it. Finally, the genetic algorithm can easily integrate additional information, without altering its stochastic properties; in this case, we add a learning component. To solve this challenge, the current work seeks to develop a new similarity-aware framework by incorporating a deep learning model to streamline the genetic algorithm. This paper introduces a hybrid architecture in which the crossover operation is preceded by a similarity assessment between parent S-boxes using a Siamese Transformer network trained for similarity learning. If the network classifies the two parents as being considerably similar, then a tailored “aggressive” crossover is triggered that purposefully adds diversity to escape local optima [9]. In aggressive crossover operations, the parents undergo prior synthesis before they are required to generate offspring. Multiple crossover points (typically, ranging from two to four) are selected, and the parents’ S-boxes are divided into segments. Approximately 50% of these segments are being substituted with arbitrary chunks, introducing diversity. These parents then further undergo a “repair step” to eliminate any redundant elements, if any. On the other hand, if the parents exhibit sufficient diversity, a standard crossover takes place. This dynamic, feedback-driven approach transforms the traditionally stochastic crossover mechanism into a targeted, intelligent operation, ensuring that computational effort is directed toward generating genuinely novel and superior offspring. The effectiveness of the proposed similarity learning-based framework is evaluated with a hybrid loss function that jointly computes Contrastive loss and Kullback–Leibler (KL) Divergence to capture geometric and distributional similarities of S-boxes embeddings. Although the suggested framework is generic, this paper addresses how it works on the S-box optimization problem as a demanding test problem to explore its effectiveness in balancing convergence and diversity. The main contributions of this paper are listed as follows:

1. *Novel Model for Boosting Genetic Algorithm Functionalities and Optimized S-box Design*: This paper proposes a similarity-aware crossover mechanism for genetic algorithms, specifically designed and evaluated for the optimization of cryptographically strong S-boxes. The approach integrates a Siamese transformer network with the Genetic algorithm’s decision-making capabilities and transitions the stochastic crossover into intelligent operation. The model ensures that crossover operations are guided by parental diversity, which is crucial for producing superior offspring. This is achieved by intelligently performing “aggressive” or regular crossover depending on the similarity (diversity) between the parents. This results in improved performance metrics and attains a nonlinearity of 110.25, which is on par with the other contemporary methods. Also, the differential uniformity of eight is achieved, which underlines the strength of the designed S-box.
2. *Novel Hybrid Loss function*: This paper presented a hybrid loss function designed by the combination of Contrastive loss and KLD (Kullback–Leibler Divergence). This novel loss function helps capture geometric distances as well as spatial similarities, thereby helping the model to achieve a higher accuracy of approximately 89% and an AUC of 0.88.
3. *Initiating a New Research Trajectory Combining Optimization Algorithms and Similarity Learning*: This paper is a step in the direction of combining the Deep learning model with the Evolutionary algorithms and enables their performance enhancement and optimization. This paper’s creation of a hybrid architecture that combines the stochastic crossover operation of the genetic algorithm with the Siamese network is a significant addition that aids in the discovery and exploitation of better solutions. This avoids traditional approaches, which offer no clear control over the entire procedure.

This paper is structured into five sections for clarity and coherence. Section 2 examines pertinent literature, summarising current methodologies and identifying deficiencies in the existing research. Section 3 delineates the suggested methodology, encompassing the framework, methodologies, and implementation particulars. Section 4 presents the results and provides a comprehensive analysis to assess the efficacy of the suggested methodology. Section 5 ultimately finishes the report by summarizing essential findings and delineating avenues for future research.

## 2. Related Works

Similarity learning is an approach of assessing the similarity between data instances and helping with the operations to be carried out in a piecewise and relational manner [10]. The concept of Similarity learning has been used extensively in recommendation systems, Image retrieval, and identity verification contexts. Apart from this, Similarity learning finds its applications in healthcare, biometric security, etc. In [11], Bello Gracia et al. explained how Similarity learning architectures use physical laws to bridge the gap between theoretical deep learning models and underlying practical explanations. Sanfins, G. et al. [12] demonstrated a pattern recognition application using Similarity learning principles by learning complex similarity patterns in data. In [13], Huang, W. et al. introduced relative comparison instead of abstract distances for a similarity learning framework. By effectively learning embeddings, the algorithm demonstrated improvement in classification and retrieval tasks. Tourad [14] discussed network architecture choices for similarity learning while demonstrating the model’s effectiveness on benchmark datasets. Chang et al. [15] leverage the k-nearest neighbors algorithm for capturing local and global patterns for better similarity assessments. Besides this, Similarity learning has also found its applications in NLP, Few-Shot learning, etc. This paradigm has transformed the field of AI and multiple other sectors. It is a well-known area of machine learning focusing on identifying similarities and differences between modalities.

Preserving diversity is a long-standing research topic in the field of evolutionary algorithms. Multi-population or island models are among the most well-known methods to preserve the diversity [16,17,18]. A population of the island model is decomposed into multiple subpopulations (islands). These subpopulations independently evolve with the occasional exchange of individuals between them. The method allows simultaneous exploration of the search space and prevents the early loss of diversity. In particular, the performance of convergence is significantly enhanced.

Cellular genetic algorithms are another class of diversity-preserving methods [19,20,21]. Individuals of cellular genetic algorithms are represented as a spatial grid, and only neighborhood individuals can interact with each other. This interaction limits the diffusion rate of the best individuals. Consequently, cellular genetic algorithms maintain diversity over generations. Spatially structured evolutionary algorithms have shown good performance in balancing exploration and exploitation.

Fitness sharing, crowding, and niching methods are additional examples of diversity-preserving methods [22,23,24]. The goal of these methods is to preserve the diversity explicitly. This implies that maintaining the heterogeneity of a population is an important design factor of an evolutionary algorithm. The similarity-based adaptive crossover proposed in this paper is another diversity-preserving method. The novelty of the proposed method is that it realizes diversity preservation in a learning manner.

In recent years, there has been an increasing focus on studying evolutionary algorithms via complex networks. For instance [25,26], evolutionary computation and swarm intelligence were studied from complex network perspectives. The hybrid evolutionary algorithm presented here, however, utilizes the results of the similarity learning for crossover and does not explore the application of the complex network analysis in the proposed model.

The application of GA for optimization and design processes is very common, and vice versa can also be true nudges towards a promising performance. Moving in that direction, Wang et al. in [27], presented a novel method of applying a genetic algorithm for optimizing S-box generation. The systematic design of S-boxes using optimization techniques has been an essential element of these studies. In [28], Msolli et al. have put forward the dynamic generation of S-boxes. It is a method to strengthen security in IoT applications by using GAs and boost cryptographic strength and flexibility. Kuznetsov et al. [29] explored non-linear substitution optimization in symmetric ciphers. They used evolutionary algorithms to improve resistance against differential and linear cryptanalysis. In another experiment, bijective S-boxes were generated by Wang et al. [30] using the GA optimization technique. They demonstrated how it enhanced the S-boxes’ nonlinearity and Differential uniformity. In addition, researchers have also combined evolutionary algorithms and neural networks to generate S-boxes with better cryptographic properties [31]. Garipcan et al. [32] explored the Particle Swarm Optimization (PSO) to enhance the S-boxes’ nonlinearity and avalanche properties. This underscores that the use of evolutionary algorithms for the design of S-boxes is an active and promising area of research.

## 3. Proposed Methodology

The proposed methodology consists of three phases: Dataset preparation—capturing data characteristics; designing and developing a model for learning similarity between two input data; and integrating the model with a genetic algorithm for guiding the crossover operation. This method preserves population diversity and mitigates premature convergence, a prevalent issue encountered by genetic algorithms. It presents an intelligent approach to direct the crossover operation in genetic algorithms, which typically depends on random or fitness-based methods [32,33].

### 3.1. Dataset Description

Our framework treats each chromosome (individual) as a candidate S-box. For each S-box, we use a permutation encoding method where each index of the permutation is the number to which each input is mapped. In this sense, the representation of the solution can be considered as a vector. However, the cryptographic properties of the S-box, such as nonlinearity, differential uniformity, and resistance to linear and differential attacks, depend on multiple non-linear relationships among several positions in the vector. In other words, two S-boxes that are similar with respect to a simple component-wise comparison of the permutation vector may have distinct cryptographic properties. Therefore, the similarity concept here has a deeper meaning than simple element-wise matching.

The model is instructed to discern the resemblance among the data points (S-boxes). Similarity learning utilizes basic Siamese networks for this objective. Siamese networks are trained on datasets comprising similar and disparate pairs (positive and negative samples). These networks acquire knowledge by recognizing analogous and disparate data points, a process referred to as maximizing the agreement [8]. The dual data points—in this case, substitution boxes—are compiled in order to train the suggested network. The dataset is compiled by incorporating both high nonlinearity S-boxes and low nonlinearity S-boxes concurrently. The selected S-boxes of the dataset are collected from prior experimental work and have robust cryptographic characteristics [33]; these serve as positive samples for the experiment. Similarly, S-boxes with low nonlinearity values also serve as positive samples and are generated using random (permutation) methods. Negative samples are generated using random S-boxes and coupled with these highly nonlinear S-boxes, so that a pair of S-boxes with diverse nonlinearity values is present. The dataset now comprises a combination of both types of S-boxes, offering substantial variation for the proposed network to learn from. Initially, the nonlinearities of the two S-boxes are computed, denoted as *n*_1_ and *n*_2_. To measure the resemblance between the two, the absolute difference between the two is calculated as shown in (1):

(1) $$L=\left|n_{1}-n_{2}\right|$$

For the specified dataset, *L* is calculated for each pair of S-boxes. The maximum nonlinearity value (*M*) is identified, and each *L* → (*L*_1_, *L*_2_,…, *L_n_*) is normalized by dividing by *M* to constrain similarity values within the range of 0 to 1, as shown in (2):

(2) $$L=\left|\frac{n_{1}-n_{2}}{M}\right|$$

Similar S-boxes exhibit a similarity score approaching 0 according to (1), while disparate ones possess a high value around 1. Reversing this behavior by deducting the similarity score from 1 produces the results depicted in (3):

(3) $$L=1-\left|\frac{n_{1}-n_{2}}{M}\right|$$

**Figure 1:**
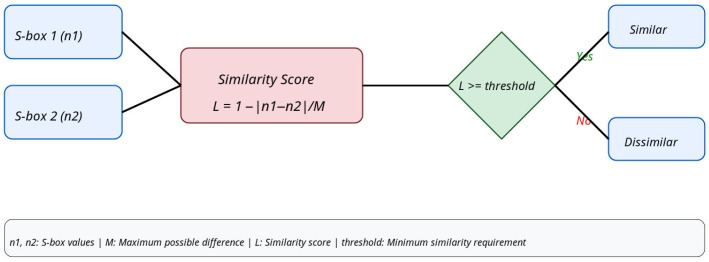
Similarity assessment of two S-boxes based on the nonlinearity difference.

This way, the dataset gets prepared with a substantial amount of diversity in it, suitable for training a Siamese network. Graphically, this process can be depicted as shown in Figure 1. The total size of the dataset prepared is 139,382 Substitution boxes, comprising approximately 69,381 dissimilar pairings and 70,001 comparable pairs. This calculation demonstrates that the data is not distorted and is appropriately balanced. The subsequent phase in data set preparation is normalization. Deep learning models perform well with normalized data. First, the mean and standard deviation of the elements in the S-boxes are calculated, subsequently subtracting the mean (μ) from the data points and dividing by the standard deviation (σ). Each S-box is standardized to a uniform format as described in (4):

(4) $$NormalizedSbox(i)=\frac{Sbox(i)-\mu }{\sigma }$$

To mitigate potential bias, S-boxes with different cryptographic properties (nonlinearity, differential uniformity, etc.) are added to the training set, so that the network will be trained on both good and bad candidates and will not adapt too much to one type of structure. Similarly, pairs of S-boxes that have different similarities are picked, so that the network will not learn to distinguish between S-boxes with respect to only one type of performance. The use of a neural network as a similarity function introduces inductive bias. This bias can be reduced by choosing the training set and the contrastive loss function. The similarity function is learned, and it evaluates the similarity between pairs of S-boxes. It does not encode any knowledge at the bit level, which may reduce the generality of the similarity, but our framework is not restricted, and more knowledge can be encoded through additional pairs of S-boxes. In any case, the learned similarity should be understood as a reasonable similarity rather than an inherent property of the S-boxes.

### 3.2. Model Preparation and Training

**Figure 2:**
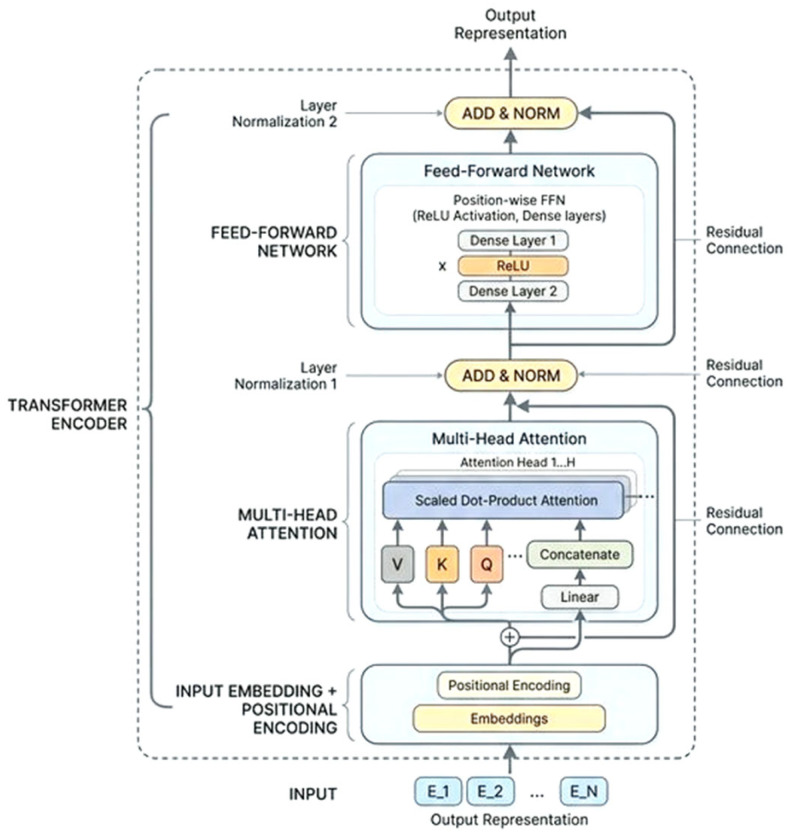
Transformer encoder architecture.

During the preparation and training phase, a model is built upon the fundamental concept of Siamese neural networks by leveraging transformer encoders to construct a network including four attention heads that employ self-attention mechanisms. Each transformer layer incorporates normalization and residual connections, with a dropout rate of 0.3 for regularization. After the transformer layers, a multilayer perceptron (MLP) with two hidden layers is implemented, each with 64 dimensions, employing ReLU activation functions and incorporating dropout between the layers as shown in Figure 2. The network culminates in a terminal linear projection that translates the MLP output back to the original input dimension of 256. A train-test split of 90 and 10 percent is used, respectively, and the dataset is analyzed in batches of 128 samples each. The loss function is a hybrid loss function that integrates contrastive loss with Kullback–Leibler (KL) divergence. The first component, Contrastive loss, is formally introduced in a seminal work by Hadsell et al. [34], which pushes the embeddings of similar inputs together and dissimilar inputs apart. Given two input points, x1 and x2, the contrastive distance is computed as shown in (5):

(5) $$d={\left\|f\left(x_{1}\right)-f(x_{2})\right\|}_{2}$$

And the contrastive loss is computed as shown in (6):

(6) $$L_{contrastive}=y.d^{2}+\left(1-y\right).max{\left(margin-d,0\right)}^{2}$$

where margin is the measurement of the minimum separation between contrastive pairs. The second component is the Kullback–Leibler loss, which is introduced in [35] by Kullback and Leibler and is computed as shown in (7):

(7) $$L_{KLD}=\sum P\left(x_{1}\right).log\left(\frac{P\left(x_{1}\right)}{P\left(x_{2}\right)}\right)$$

The final objective function (the proposed combined Contrastive + KLD loss function) is defined as (8):

(8) $$L_{total}={\alpha .L_{contrastive}+\left(1-\alpha \right).L}_{KLD}$$

where KLD loss is the Kullback–Leibler Divergence loss, and that α = 0.5 balances the two loss components. The hybrid loss combines contrastive loss, which measures the Euclidean distance between paired embeddings, with KL divergence, which evaluates the relative entropy between output distributions. The KL divergence loss (KLD-Loss) quantifies the relative entropy between two probability distributions. *P*(*x*_1_) represents the probability distribution of the features associated with the initial input, and *P*(*x*_2_) denotes the probability distribution of the features associated with the second input. The values of *P*(*x*_1_) and *P*(*x*_2_) are derived by applying the SoftMax function to the network outputs. The summing encompasses all dimensions of the output space. KL divergence helps ensure the following key aspects:

- **Distribution Alignment:** Maintaining the overall distribution of features derived from the data points (S-boxes) is essential. The data structure holds equal significance to the data itself. The Contrastive Loss quantifies the absolute distance between two data points, but the KL divergence guarantees the consistency of feature distributions. This is especially crucial for cryptographic S-boxes, because the relative relationships among features may be as significant as their absolute values.
- **Preserving Information:** The output embeddings, when data diversity is inadequately represented, are penalized by the KL divergence. This guarantees the capture of essential data features, hence preserving the information.
- **Regularization Effect:** The Contrastive loss consolidates comparable pairings while separating dissimilar pairs, whereas KL divergence maintains the integrity of the underlying probability distribution of the clusters.
- **Improved Robustness:** By incorporating both geometric distances (by contrastive loss) and distributional similarities (via KL divergence), the model exhibits more resilience to diverse variances in the input S-boxes.

A hyperparameter labeled as α is incorporated to equilibrate the influence of both loss functions. Setting α to 0.5 facilitates the equilibrium between distance and distribution-based losses, hence aiding in the capture of both structural and probabilistic commonalities among the S-boxes. The AdamW optimizer [36] is used for optimization, setting the learning rate to 1 × 10^−6^ and the weight decay to 1 × 10^−5^. The learning rate is modified dynamically through the OneCycleLR scheduler [37]. Many regularization approaches are leveraged, including gradient clipping [38] with a maximum norm of 0.5, which adjusts gradients when their norm goes above this limit. If ${\left\|g\right\|}_{2}>0.5$, then the gradient ***g*** is normalized according to (9).

(9) $$g=0.5.\frac{g}{{\left\|g\right\|}_{2}}$$

**Figure 3:**
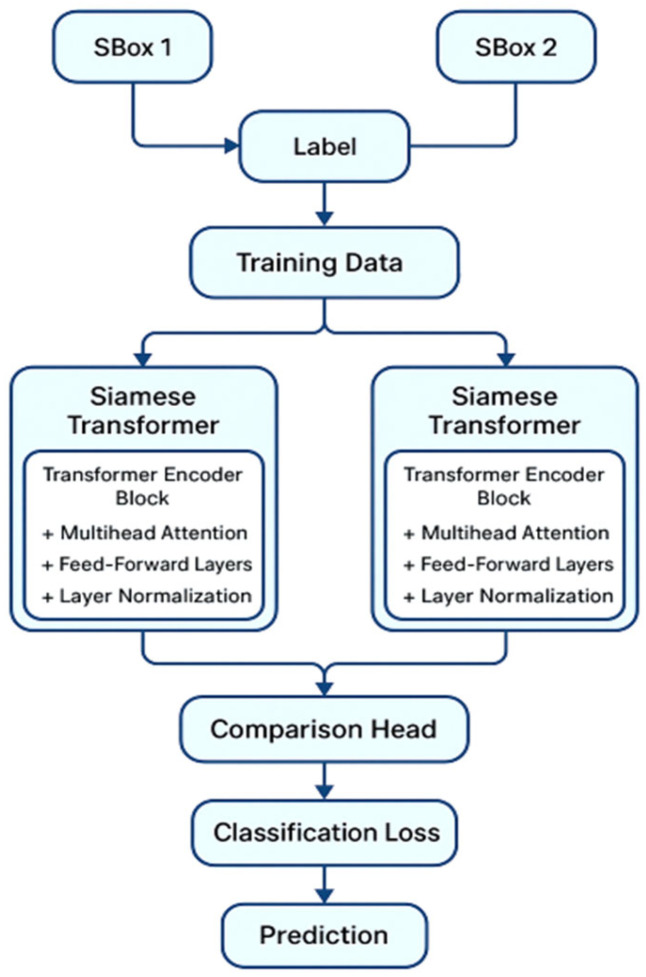
Network architecture of the Siamese model.

The network architecture has two transformer blocks, each employing a feed-forward dimension of 128 and four attention heads, as shown in Figure 3. To ensure a fair comparison between the different model variants and guarantee reproducibility, we utilize a random seed value of 42 for every experiment, including data manipulation, model instantiation, and genetic algorithm processes. It is crucial to note that every experiment (vanilla, contrastive-guided, KL-guided, and the proposed combined model), begins with the same S-box population, and the experimental setup ensures the reproducibility of the proposed similarity-guided crossover approach. Model checkpoints are preserved according to the highest validation accuracy attained during training. The maximum number of training epochs is established at 500; however, early halting frequently activates prior to this threshold, thereby averting overfitting. Model evaluation is conducted through validation accuracy for paired samples, computed as per (10):

(10) $$Accuracy=\frac{TP+TN}{TP+TN+FP+FN}$$

Although learning a transformer-based Siamese network adds an extra learning task to the Evolutionary Algorithms (EA), it is important to emphasize that the proposed approach does not replace the underlying genetic algorithm but rather augments it. The model is used only to predict the similarity of individuals, and it is not constrained to strongly control the EA. Concretely, the stochastic behavior of the crossover and mutation operations is preserved. The boundary between similar and dissimilar pairs is learned softly, and it is used to suggest the selection of parents, instead of forcing the EA to evolve in a deterministic way. This allows us to preserve the power of artificial evolution while endowing it with the knowledge of the structural relationships between individuals learned by our model.

Although the learning approach introduces a certain loss of interpretability with respect to purely symbolic methods, it allows for a more adaptive and context-dependent search. The proposed framework, therefore, represents a hybrid approach in which learning-based guidance complements, rather than distorts, the principles of artificial evolution.

### 3.3. Guiding Crossover in Genetic Algorithm

**Figure 4:**
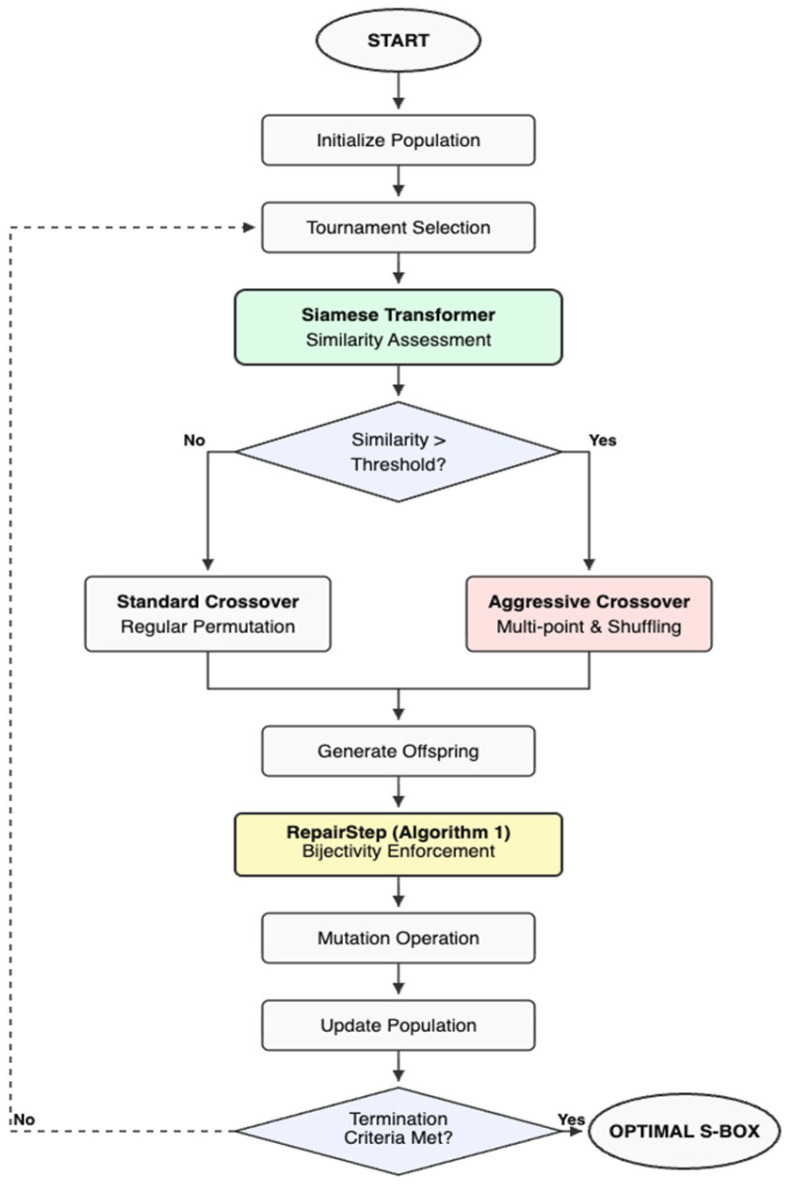
Flowchart of proposed GA with model-guided crossover.

This is the third phase of the proposed architecture, where the integration of the designed similarity network into the decision-making process of the genetic algorithm takes place. The proposed approach involves integrating a pre-trained Siamese Transformer network into the decision-making process of the genetic algorithm, aimed at enhancing the efficiency of crossover operations. This innovative method improves the algorithm’s cognitive capabilities compared to traditional techniques. The network assesses the similarity between parent S-boxes by analyzing their cryptographic functions and structural patterns, as shown in Figure 4. Unlike conventional genetic algorithms that apply crossover universally across all parent pairs, this approach uses similarity scores to selectively guide crossover, thereby preventing ineffective operations when parents are too alike.

The proposed approach involves probabilistically identifying crossover points within S-boxes. When dealing with dissimilar S-boxes, a standard crossover method is applied that permutes elements while maintaining stability and promoting diversity, thereby generating viable offspring. In cases where S-boxes are highly similar, an aggressive crossover strategy is employed.

**Figure 5:**
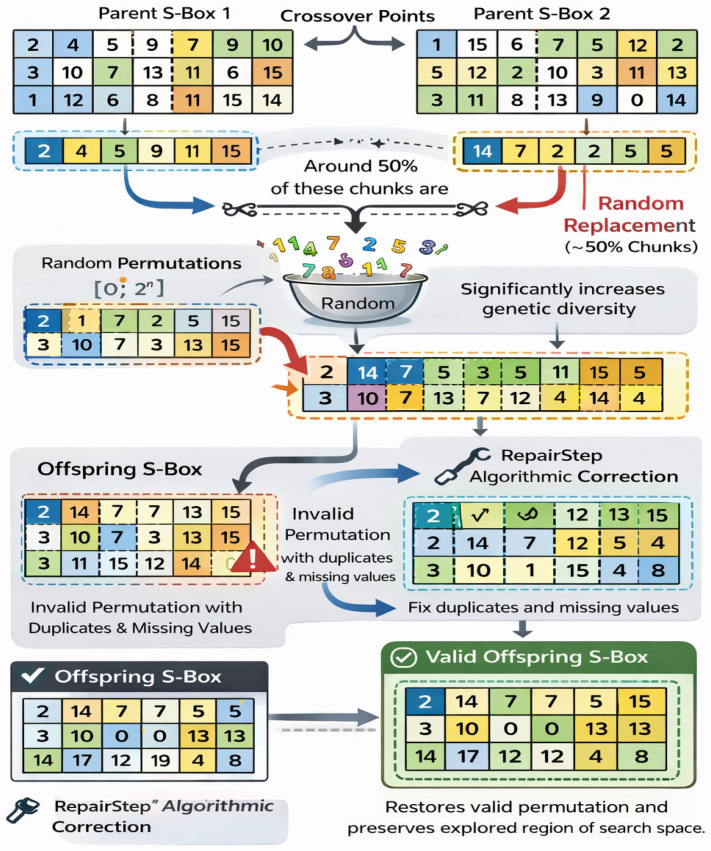
Visual representation of Aggressive crossover and RepairStep in the framework.

Aggressive crossover is a strategy introduced to handle similar parents. Since similar parents do not have much diversity to offer, we devised a strategy to induce diversity in the offspring. In this strategy, instead of choosing a single crossover point, multiple crossover points are selected, and parent S-boxes are divided into chunks. Around 50% of these chunks are being replaced randomly with the chunks consisting of elements, designed through random permutations ($\in \left[0,\;2^{n}\right],\;where\;n\;is\;the\;size\;of\;S-box$). This targeted disruption purposefully induces significant genetic diversity into the offspring to forcefully steer the population away from premature convergence and stagnant local optima. Because this intense replacement inherently violates the bijectivity required of a valid cryptographic S-box by introducing duplicate and missing elements, the operation is immediately followed by a “RepairStep” algorithmic correction that replaces duplicates with randomly selected missing values, thereby restoring a valid permutation while preserving the newly explored region of the search space. The Repair Step is implemented after aggressive crossover to rectify invalid offspring S-boxes that exhibit duplicate and missing elements. It detects duplicate values and substitutes the additional instances with randomly chosen missing values from the acceptable range. This procedure reinstates the bijective (permutation) characteristic necessary for a legitimate cryptographic S-box. Simultaneously, it maintains the diversity generated during crossover by making modest modifications to the altered structure. Figure 5 presents the working of the Aggressive crossover operation and RepairStep in a detailed manner.

Aggressive crossover introduces a degree of randomness by replacing crossover points with random elements to increase variability. The method relies on a threshold value that determines whether a standard or aggressive crossover is used. If the values exceed the set threshold, this initiates an aggressive crossover. If the value is under or equal to the threshold value, the crossover performed is the standard one. To rectify any violations in the reproduction of offspring, a correction mechanism is put in place. This mechanism ensures that the resulting S-boxes remain valid permutations, thereby eliminating the possibilities of any vague generations.

This experiment aims to prevent premature convergence by monitoring the high-performing Substitution-boxes and tracking the population diversity. These metrics also inform about the early termination as and when further improvement slows down, thereby optimizing computational efficiency. Employing the pattern recognition capabilities of the Siamese Networks helped balance the exploitation and exploration of solutions, paralleled by the optimization strengths of the genetic algorithm. This approach, bypassing the traditional, uncontrolled mechanisms that solely depend on the mathematical constructs, helped the autonomous generation of the S-boxes, which are cryptographically sound. This framework is adaptive for other cryptographic primitives as well, thereby improving the search efficiency and quality of the solutions generated.

**Objective Functions and Fitness Evaluation**: The Substitution boxes are evaluated based on two essential cryptographic properties, namely nonlinearity (NL) [39] and differential uniformity (DU) [40]. The nonlinearity is calculated with the help of Walsh-Hadamard Transform (WHT) [41], defined as follows in (11):

(11) $$NL\left(f\right)=2^{n-1}-\frac{1}{2}max(\left|WHT(f)\right|)$$

where *n* is the size of the S-box, it is equal to 8 for an 8 × 8 S-box. A higher score of NL facilitates the induction of high nonlinearity in the system and mitigates the linear cryptanalysis. The differential uniformity (DU) follows the formulation given in (12). A lower score of DU is desirable for vindicating the differential cryptanalysis.

(12) $$DU(S)=\max\delta \left(\alpha ,\beta \right)|\alpha \neq 0,\beta \in GF(2^{n})$$

where $\delta \left(\alpha ,\beta \right)$ is defined as follows:

$$\delta \left(\alpha ,\beta \right)=\left|\left\{x|S\left(x+\alpha \right)+S\left(x\right)=\beta \right\}\right|$$

The optimization of cryptographic S-boxes is by nature a multi-objective problem (e.g., maximizing nonlinearity and minimizing differential uniformity). This study, however, takes a single-objective stance by combining the objectives into a fitness function. This is primarily because the framework needs a well-defined objective function for the evolutionary algorithm as well as for the Siamese Transformer model, and the latter needs a single scalar loss function to compute loss and update parameters. Dealing with multiple Pareto fronts in the context of the learning framework is non-trivial. The combination of objectives is done under the assumption that, e.g., a small increase in differential uniformity can be compensated for by a larger increase in nonlinearity. Clearly, this does not allow for the exploration of the full Pareto front of optimal S-boxes but greatly simplifies the optimization process and convergence to good S-boxes. A proper multi-objective treatment would necessitate dealing with a set of Pareto-optimal S-boxes both in the context of the evolutionary algorithm and the learning framework. Thus, the single objective function is defined as (13).

(13) $$objective\left(S\right)=NL\left(S\right)+(256-DU\left(S\right))$$

The combined fitness function is defined as (14).

(14) $$fitness\left(S\right)=NL\left(S\right)+(2^{n}-DU\left(S\right))$$

The population diversity is tracked by using average pairwise differences as calculated according to (15).

(15) $$diversity=mean\left(\frac{\sum (S_{i}\neq S_{j}}{\left|S\right|}\right)\;\forall \;pairs\;i,j$$

where *S_i_*, *S_j_* are S-boxes in the population. The proposed algorithm is initialized with a population size of 100 and undergoes 500 generations. To retain the best offspring produced from each generation, we employ a mechanism called Tournament selection. In this process, five participants compete, and the one with the best fitness score is retained and gets to pass down its traits. To compute the similarity score of potential parents, the Siamese network is employed as (16).

(16) $$similarity(S_{1},S_{2})=\frac{1}{1+d}$$

where *d* is the Euclidean distance defined as:

$$d={\left\|f\left(S_{1}\right)-f(S_{2})\right\|}_{2}$$

The integration of the Siamese Model into evolutionary algorithms introduces an inherent trade-off between performance and explainability. While traditional genetic algorithms are fully interpretable and allow direct tracing of evolutionary steps, the inclusion of a neural similarity model reduces the transparency of the decision-making process that guides crossover operations. In particular, the learned similarity function operates as a data-driven approximation, making it difficult to interpret why certain individuals are considered similar or dissimilar explicitly.

However, it is important to note that the overall evolutionary process remains fully traceable. All genetic operations, including selection, crossover, and mutation, are explicitly defined and recorded, and the role of the neural model is limited to providing a similarity score that influences, but does not replace, these operations. Thus, while local interpretability at the similarity estimation level is reduced, global traceability of the optimization process is preserved.

This trade-off is consistent with a broader trend in hybrid and neuro-symbolic systems, where improved adaptability and performance are achieved at the cost of reduced transparency in certain components. In this work, the performance gains in terms of improved diversity preservation and convergence behavior are considered to justify this compromise, while acknowledging that enhancing the interpretability of the learned similarity function remains an important direction for future research.

## 4. Experimental Results

This section presents a detailed analysis of the working of the proposed methodology. The overall focus is two-fold: tracking the performance of the Siamese network in similarity learning and assessing the efficacy of the genetic algorithm in optimizing the S-box design, guided by the Siamese network. Each phase of the experiment is comprehensively analyzed, ensuring a thorough evaluation of the proposed approach. To ensure that our experiments are reproducible, we set all random seeds to deterministic values. All runs of each configuration start with the same initial population, guaranteeing that comparisons between the different model variants are consistent and fair in terms of the impact of the proposed similarity-guided crossover operation.

### 4.1. Performance Analysis of the Siamese Network

To study the work and performance of a transformer-based model, extensive experiments were conducted with hyperparameter fine-tuning, architecture scaling, and different loss functions. To begin with, the process started with a baseline model with a standard transformer configuration including six encoder layers, a hidden size of 512, eight attention heads, and a dropout rate of 0.1. The baseline model is trained for 50 epochs with a learning rate of 0.001 and a batch size of 32, using Contrastive Loss as the objective function. After finishing the training, the final metrics were as follows: final training loss of 0.25, a validation loss of 0.27, and an accuracy of 85%. From these metrics, it could be inferred that the model effectively captured the core patterns in the data.

**(i) Hyperparameter tuning and Architectural Scaling**: To evaluate the efficacy of the Siamese network in learning similarity among S-boxes, an experiment with fine-tuning the hyperparameters is carried out; Reducing the learning rate to 0.0005 and the batch size to 64, accelerating the model learning. The metrics attained are a training loss of 0.23, a validation loss of 0.26, and an accuracy of up to 87%, respectively, indicating better generalization and smoother updates. Subsequently, the architecture is enhanced by increasing the number of encoder layers from 6 to 12 and increasing the attention heads from 8 to 16, reverting the learning rate and batch size to the original. This shift gave the model a real boost, dropping the training loss to 0.21, the validation loss to 0.24, and improving the accuracy to 89%, as shown in Table 1. Although this setup really improved the performance, it also meant that the proposed algorithm became resource-intensive, thereby raising the need for careful regularization to avoid overfitting.

**(ii) Loss Function Variations**: It has also been observed how different loss functions affect model training, which is of equal importance.

**Figure 6:**
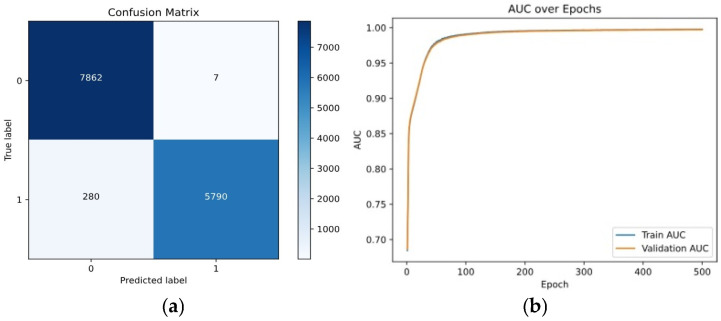
Performance for Contrastive Loss: (**a**) Confusion matrix, (**b**) AUC curve.

**Figure 7:**
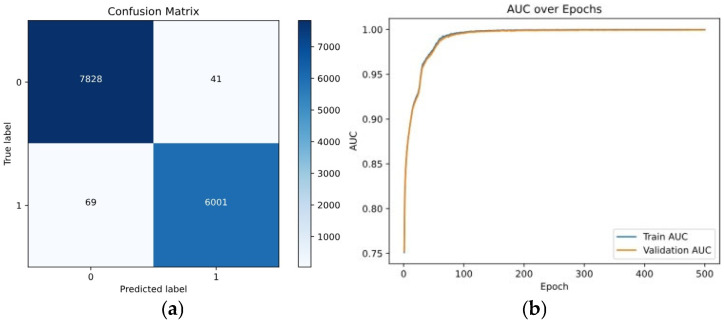
Performance for KL Divergence Loss: (**a**) Confusion matrix, (**b**) AUC curve.

**Figure 8:**
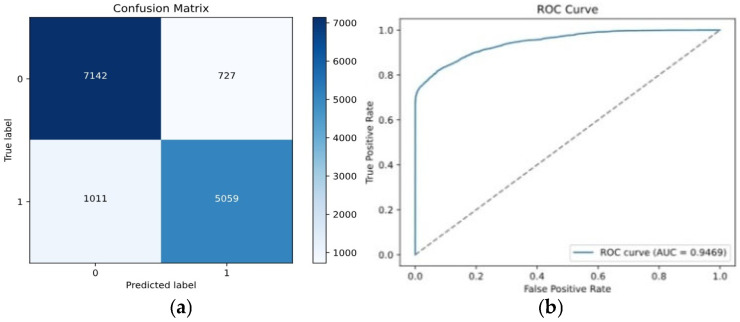
Performance evaluation for Contrastive-KL Loss: (**a**) Confusion matrix; (**b**) ROC.

- *Contrastive Loss Trends*: The contrastive loss function, which minimizes intra-class variance and maximizes inter-class variance, is examined over training iterations. When using Contrastive Loss alone, the model had a training loss of 0.28, a validation loss of 0.30, an accuracy of 82%, and an AUC score of 0.85, establishing a solid baseline for binary classification. Figure 6 shows the Confusion Matrix and AUC curve, illustrating effective class separation but indicating room for improvement.

- *KL Divergence Loss Trends*: The KL divergence plays a critical role in preserving the distribution of features by aligning the embedded representations with a reference distribution. As the network attempted to adapt in the latent feature space, fluctuations were initially seen. Performance was slightly improved when the KL Divergence loss function was integrated with Contrastive loss. The training loss got reduced to 0.26, the validation loss dropped to 0.28, accuracy shot up to 84%, and the AUC score increased to 0.87. The updated Confusion Matrix (Figure 7a) and AUC Curve (see Figure 7b) show that incorporation of KL Divergence helped improve true positive rates and made the different classes easier to separate.

- *Novel Contrastive + KL Loss Function*: Combining Contrastive and KL loss functions, moderated by the parameter α, resulted in effective convergence through training epochs. The unified loss delivered superior results, with a training loss of 0.24, a validation loss of 0.27, an accuracy of 89%, and an AUC of 0.88. These findings indicate the robustness of the Contrastive-KL loss function in capturing detailed features and improving class differentiation, though parameter tuning may optimize performance across different scenarios. Figure 8 displays the confusion matrix and ROC curve corresponding to this combined loss.

**Figure 9:**
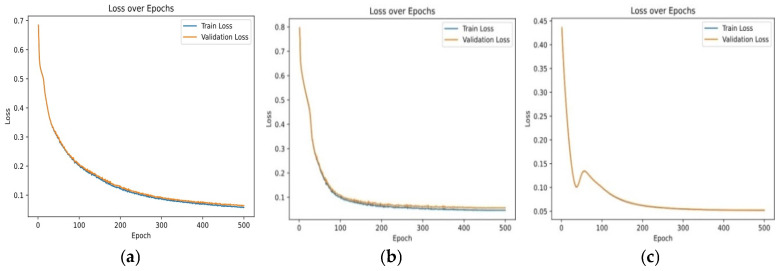
Loss over epochs plots for: (**a**) Contrastive Loss; (**b**) KL Divergence Loss; (**c**) Contrastive + KL Loss.

The comparison of the loss curve is illustrated in Figure 9, showing each loss function’s performance. Table 2 demonstrates the performance comparison of loss functions. These results show that the new loss function not only helps the model learn finer details better but also makes it more likely to perform well on new, unseen data.

### 4.2. Boosting Genetic Algorithm with Siamese Network

In this section, the impact of incorporating a Siamese network into a genetic algorithm (GA) on the quality of S-boxes generated is studied. The comparison of the proposed algorithm with that of the traditional crossover method (single-point crossover method) by using a similarity-guided, adaptive crossover strategy is carried out. This method has changed over 500 generations through experimentation with various population sizes of 100, 500, and 1000 individuals. The vanilla GA (without modifications), the Contrastive-guided (using Contrastive loss), the KL-guided (using KL divergence loss), and the suggested combined Contrastive-KL-guided approach have all been assessed. Table 3 provides a brief definition of each variant. In all these variants, the key metrics, namely nonlinearity (NL), differential uniformity (DU), and diversity, are tracked to evaluate the performance and efficacy of the proposed model.

**(i) Results across population sizes**: For every population size of 100, 500, and 1000, the algorithm evolved over 500 generations. For each generation, the parameters being recorded are nonlinearity (NL), differential uniformity (DU), and population diversity scores. The main aim is to evaluate the improvements in terms of cryptographic soundness, convergence, and robustness of the algorithm.

**Figure 10:**
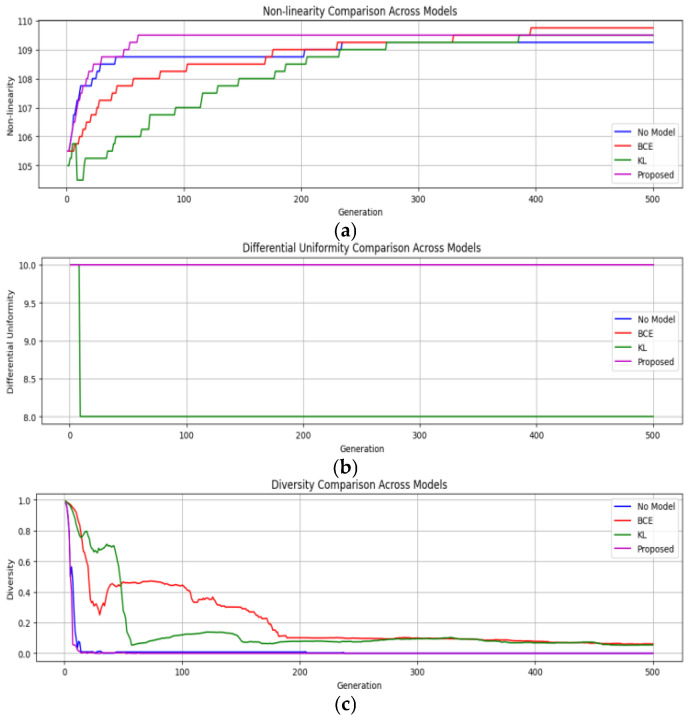
Performance across all the models for population size = 100 (**a**) Nonlinearity vs. Generation; (**b**) Differential Uniformity vs. Generation; (**c**) Diversity vs. Generation.

**Figure 11:**
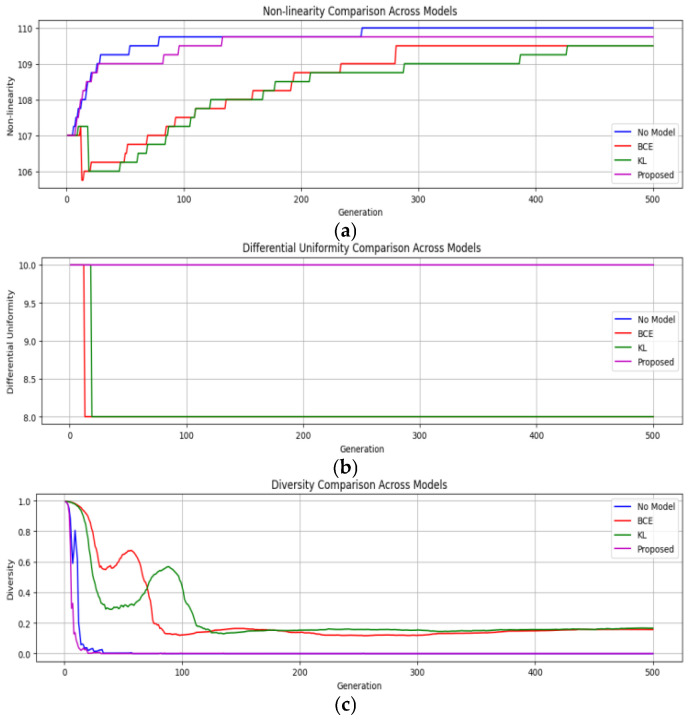
Performance across all the models for population size = 500 (**a**) Nonlinearity vs. Generation; (**b**) Differential Uniformity vs. Generation; (**c**) Diversity vs. Generation.

**Figure 12:**
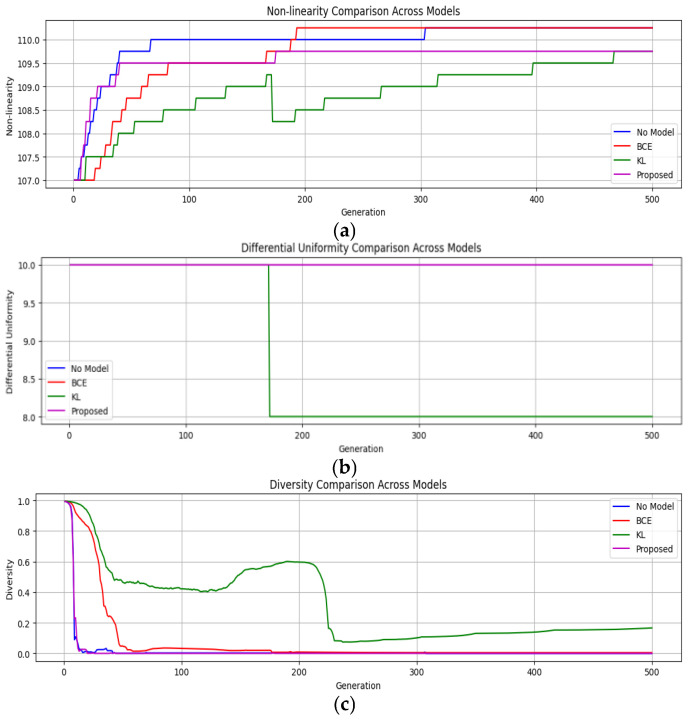
Performance across all the models for population size = 1000 (**a**) Nonlinearity vs. Generation; (**b**) Differential Uniformity vs. Generation; (**c**) Diversity vs. Generation.

- *Population Size* = 100: The standard GA converged by the 200th generation, stabilizing the improvement curve after achieving a decent score for nonlinearity (NL), but demonstrated low diversity as shown in Figure 10a,b. In another model, in which Contrastive loss has been used to guide the GA, the observations were promising. The model could choose the crossover points strategically early on (generations 1–150), which resulted in an increase in NL score by approximately 7 to 9%. On the other hand, the KL-guided model used a softer, probabilistic approach to similarity. It did not show improvements initially, but kept improving steadily, maintaining high diversity and surpassing the Contrastive Model around the 400th generation. The proposed combined Contrastive-KL loss model got quick gains like the Contrastive one, but also kept the diversity benefits from KL divergence, keeping momentum across all 500 generations, delivering the best cryptographic results, improving average nonlinearity by about 12% over the baseline, as shown in Figure 10a.
- *Population Size* = 500: Initially, the baseline GA maintained a decent level of diversity but stagnated around generation 200, as shown in Figure 11a,b. The Contrastive-guided approach managed to enhance NL and reduce DU early in the process, although diversity experienced a decline between generations 250 and 350 due to a tendency to favor similar parent solutions. The KL-divergence-guided model preserved an initial diversity level, allowing for more sustained exploration, maintaining approximately 15% higher diversity score than the Contrastive model during the critical middle phase. Even though this meant a slightly slower start in convergence, it avoided getting stuck at premature convergence, progressing smoothly in fitness up to generation 500, preserving diversity, and allowing cryptographic configuration. The Contrastive-KL model outperformed both Contrastive and KL individual models for this population size, as it consistently achieved the highest final NL scores and the lowest DU, showing a better balance between aggressive crossover and staying adaptable. Also, it kept a diversity score above 0.4 for all generations, twice as high as baseline from getting stuck or drifting aimlessly, making it reliable and consistent, as shown in Figure 11c.

- *Population Size* = 1000: The baseline GA benefited from a higher initial diversity, which slowed the convergence process but lacked the strategic crossover mechanisms needed for further improvement. The Contrastive-guided model outperformed the baseline by approximately 300th generations, as illustrated in Figure 12a. Due to the population’s size, its tendency to be aggressive did not reduce diversity, as there was enough variety to prevent rapid homogenization. The KL divergence approach provides a stabilizing effect in this larger population. As demonstrated in Figure 12c, the KL divergence model maintained a higher diversity (approximately 5%) compared to baseline and Contrastive models. The proposed combined loss model surpassed other models in terms of the soundness of the cryptographic properties of generated S-boxes and diversity stability, as shown in Figure 12. Despite the modest numerical improvements, the combined loss model showed 4–6% better metrics than the baseline and other models. This indicates that in large populations where diversity is already high, the effectiveness of similarity-based crossover reduces, showing diminishing results, as depicted in Figure 12c. But it showed lower variability over the generations, and the consistent improvement in S-box properties demonstrated its value well at larger scales.

The similarity-guided adaptive crossover framework appears to perform well based on the test results. It further improves performance when combined with model-driven loss optimization. In the different experiments carried out with different models, the Contrastive-guided model made strategic choices and produced good results early on, but it didn’t perform well on the diversity front. On the other hand, the KL divergence model helped maintain a more varied population, but the time taken to converge was substantial. The combined Contrastive + KL approach effectively balanced this trade-off between premature convergence and diversity, producing great results for smaller population sizes and steadily maintaining the improvement trajectory in large population sizes as well. This method improved the overall performance and lowered fitness variability by up to 20%.

**(ii) Statistical Comparison of Models across Population Sizes**: A thorough analysis of the proposed new similarity-driven adaptive crossover method was carried out by testing it against the standard genetic algorithm (GA) with different population sizes (100, 500, 1000) across the four variants namely (a) vanilla Genetic algorithm, (b) a model guided with Contrastive loss, (c) KL divergence, and (d) combined approach using Contrastive and KL, respectively. The metrics, which show cryptographic soundness, include nonlinearity, differential uniformity, and diversity of the population, were tracked over 500 generations. This section provides a comprehensive analysis of the best metrics, summarizing their average and variability, which helps to understand the working, stability, and efficacy of each approach. For each population size (100, 500, and 1000), the results are shown in Table 4, Table 5 and Table 6, respectively.

For a population size of 100, as shown in Table 4, the Contrastive-KL model attains the highest average nonlinearity score of 109.3460 and a diversity measure of 0.0099, matching a Diversity Uncertainty (DU) value of 10.0, comparable to that of standard and Contrastive alone, indicating a balanced approach between swift advancement and exploratory potential. When considering a size of 500, as detailed in Table 5, the basic Genetic Algorithm exhibits commendable initial diversity and nonlinearity, with a mean of 109.7395, yet it does not improve its DU, which remains steady at 10.00, unlike the Contrastive and KL-enhanced models. The Contrastive + KL model approaches similar levels of nonlinearity (109.5395), maintains a diversity of 0.0119, and displays the lowest standard deviation of 0.4778, emphasizing its robustness. However, its DU of 10.00 may underrepresent the model’s full capacity in longer-term or extended scenarios. At the largest population size of 1000 in Table 6, the baseline GA benefits from a bigger genetic pool and achieves the highest nonlinearity of 109.9595. Still, the proposed model stays competitive with 109.5815 in nonlinearity and the lowest diversity of 0.0165. It also maintains perfect differential uniformity at 10.00. The proposed model also has the lowest standard deviation in nonlinearity at 0.4066, demonstrating its robustness across multiple runs.

Overall, these findings lend support to the qualitative analysis. The proposed Contrastive + KL loss effectively combines Contrastive loss’s swift convergence with KL’s capacity for maintaining diversity, resulting in enhanced cryptographic properties. The Siamese Transformer assesses S-box similarity to assist more intelligent crossover decisions, striking a balance between exploration and exploitation to ensure stable evolution. These benefits are most evident in smaller populations (100 and 500), where they lead to important improvements, yet remain valuable in larger populations (1000), which tend to offer greater diversity but less guided optimization. This hybrid approach consistently produces reliable S-boxes characterized by high nonlinearity and optimal differential uniformity, representing a meaningful progression in AI-driven cryptographic design. During experimentation, a comprehensive evaluation of all four variants has been carried out across three population sizes: 100, 500, and 1000. However, to assess the potential and the ceiling of cryptographic strength of the proposed framework, an extended analysis was carried out on a population size of 120 over 1000 iterations, achieving a nonlinearity score of 110.25, a DU of 8, and a diversity measure of 0.993, as shown in Table 7. The resulting optimized S-box, as shown in Table 8, is a bijective S-box, making it highly suitable for cryptographic applications. The obtained results surpass the standard genetic algorithm, which records a DU of 10. The differential uniformity of value eight significantly strengthens the resistance against differential cryptanalysis. It is important to note that standard GA does not optimize beyond the value of 10, as it tends to stagnate. Furthermore, the nonlinearity increased in parallel with a decrease in DU, which demonstrated the balance between the two trajectories.

To assess the security strength of the proposed optimized S-box, a comparative study is carried out with the traditional GA based methods and other state-of-the-art S-box generation methods, as shown in Table 9, using the most desirable S-box security metrics, namely the nonlinearity and differential uniformity. As discussed, the proposed framework generates an optimized S-box that attains a mean nonlinearity score of 110.25, aligning with the lowest DU value of 8, and notably excels with a diversity of 0.993, exhibiting reliable convergence without premature uniformity. The proposed S-box demonstrates improved cryptographic strength compared to existing methods using genetic algorithm investigated in [29,42], reinforcement method using hash functions presented in [32], high nonlinear S-box designing using I-Chings operators in [43], a transfer-function assisted metaheuristic and booster algorithm studied in [42], an S-box generation method using mutation optimization in [44], a DC Generative adversarial network based S-box design method presented in [45], a multi-objective optimization based optimal S-box generation method given in [46], and a Roulette wheel based Social Network Search algorithm for strong S-box design proposed in [47]. Hence, it is evident that the proposed method offers great credibility for augmenting the performance of the genetic algorithm for producing optimized S-boxes. The proposed methodology stands out due to the innovative use of the Siamese Transformer network’s advanced S-box comparison mechanisms, which assist more knowledgeable crossover decisions. Unlike conventional genetic algorithms or hybrid approaches that incorporate chaos theory, this method achieves a careful balance between exploration and exploitation. As a result, it attains high levels of nonlinearity, maintains consistent differential uniformity (DU), and exhibits high diversity. Furthermore, by integrating deep learning techniques, the approach greatly improves the effectiveness of designing strong cryptographic S-boxes, establishing itself as a preferred choice for secure cryptographic components.

### 4.3. Computational Complexity and Runtime Analysis

It is a possibility to assume that the Siamese Transformer incurs significant computational overhead; our runtime analysis indicates that the evaluation takes place only once every crossover occurrence. In S-box optimization, the primary computational constraints are the cryptographic fitness evaluations, particularly the calculation of the Walsh-Hadamard Transform for nonlinearity and the creation of XOR-sum distribution tables for differential uniformity.

Empirically, given a population size of 100 using NVIDIA 4060Ti, INTEL i9, the baseline Standard GA necessitated an average of 1.42 s for each generation. The proposed framework necessitated an average of 1.48 s for each generation. This indicates that the incorporation of the Siamese Transformer adds a minimal overhead of only 4.2% to the overall cryptographic optimization endeavor. The substantial advantages in avoiding local optima and identifying superior cryptographic settings significantly surpass the 4.2% increase in runtime. These findings unequivocally demonstrate the scalability and usefulness of the proposed framework. To illustrate, running the complete genetic algorithm for 500 generations on a population of 100 takes around 710 to 740 s (≈12 min) for the existing GA and around 740 to 780 s (≈12 to 13 min) for the proposed scheme on a system equipped with an Intel i9 processor and NVIDIA RTX 4060Ti GPU. For a larger population size (say, 500 and 1000), the total execution time scales up significantly, taking up to approximately 1 to 2 h. This increase in runtime is directly proportional to the population size, primarily due to the higher number of fitness evaluations and similarity computations. The computational cost is mainly associated with the evaluation of cryptographic fitness functions, such as the Walsh–Hadamard transform and differential uniformity, both of which are sensitive to changes in population size and the S-box size. Running numerous separate experiments for each setting is challenging because evaluating fitness repeatedly can be expensive, especially for larger populations. Therefore, results are provided from a representative run using the same seed in the current study to ensure reproducibility of results. The general trends observed from different population sizes and model variants, however, are stable and consistent with each other.

## 5. Conclusions

This paper shows how combining deep learning with genetic algorithms (GAs) can really improve the way to carry out the systematic design of cryptographic S-boxes. In this work, a comparative study of the standard genetic algorithm with the more advanced versions guided by deep learning models using different loss functions like Contrastive loss and Kullback–Leibler (KL) divergence, and the proposed combination of both Contrastive and KL, is performed. The results demonstrated that the proposed approach, which uses a Siamese Transformer network to measure similarity and guide the crossover adaptively, demonstrates strong performance and surpasses the traditional approaches. The proposed model achieved a high nonlinearity of 110.25 with a DU of 8, and very high diversity (0.993) across different population sizes. By combining the deep learning approaches with the evolutionary strategies, this paper addressed a very critical issue of slow convergence. This also helped address the challenge of less effective solutions of conventional GAs, thereby nudging the research in a different direction. Despite promising results in producing S-boxes with good properties, the proposed approach needs to be tested on other search areas to evaluate its universality.

The presented framework is a significant step towards improving the construction of cryptographic S-boxes using similarity-based genetic algorithms; however, there are still some areas where it can be improved. Firstly, a full-fledged statistical analysis of the random process of the genetic algorithm is to be carried out. In the present work, the reproducibility is guaranteed by fixing the seed for random number generation, and the sum of metrics over generations is presented. In the future, this analysis will be further strengthened by carrying out several independent runs with different random seeds. This will facilitate a thorough analysis of the robustness, stability, and statistical significance of the scheme. Multi-objective optimization algorithms can be used in the future to optimize more than one cryptographic property simultaneously. It is also possible to improve the crossover point selection based on adaptive similarity score and different similarity learning models. The approach is not restricted to cryptographic S-boxes alone; it is very well applicable to various other cryptographic primitives and optimization problems. Moreover, a comparison with other commonly used methods to preserve diversity, such as island models or cellular genetic algorithms, will be carried out as part of the future work.

The ability of Siamese networks to enable adaptive crossover decisions opens the door for future research into multi-objective optimization, experimenting with different similarity thresholds, and applications beyond S-boxes in security domains. Overall, the findings emphasize the exciting potential of merging AI with evolutionary algorithms, paving the way for innovations in augmenting the optimal performance of meta-heuristics for different areas of applications.

Future research may also consider the use of complex network concepts for modeling and analyzing the interactions of populations in our approach, which may help to better understand convergence and diversity in theoretical terms. Future work also includes a full statistical evaluation by running these experiments on several different independent executions for different random seeds and then analyzing the performance and the variance for our different framework configurations.

## Figures and Tables

**Table 1:** Performance comparison across model variants.

Model	Encoder Layers	Train. Loss	Valid. Loss	Accuracy
Baseline	6	0.25	0.27	85
Hyperparameter Tuned	6	0.23	0.26	87
Architectural Scaling	12	0.21	0.24	89

**Table 2:** Performance comparison of Loss functions investigated in the proposed methodology.

Loss Function	Train Loss	Val Loss	Accuracy	AUC
Contrastive loss	0.28	0.30	82%	0.85
KL Divergence loss	0.26	0.28	84%	0.87
Contrastive + KL loss	0.24	0.27	89%	0.88

**Table 3:** Description of four model variants of the Genetic algorithm investigated.

Model Variant	Definition
Vanilla Genetic Algorithm (No Model)	This is the standard form of genetic algorithm where the crossover operation is not governed by any intelligent mechanism but is rather based on traditional, often random or fitness-based mechanisms. The main drawback of this approach is that it fails to utilize the local minima.
Contrastive-loss Guided Model	In this variant, GA is combined with the Siamese network with Contrastive loss as the loss function. The incorporation of the Siamese network helps in assessing geometric similarity between the two parents and guides a strategic crossover between the two.
KL Divergence loss Guided Model	This variant of GA has Kullback–Leibler Divergence loss, and the network tries to minimize it, which results in a high population diversity.
Combined (Contrastive + KL) loss Guided Model	The proposed adaptive GA is guided by a hybrid loss function that combines both Contrastive and KL Divergence, thereby balancing the benefits of fast convergence and robust diversity maintenance.

**Table 4:** Performance statistics for population size = 100.

Metric	No Model	Proposed		
		Contrastive	KL	Contrastive + KL
Nonlinearity Mean	108.947	108.884	108.3095	109.3460
Nonlinearity Std. Dev.	0.4984	0.9135	1.3724	0.5414
DU Mean	10.00	10.00	8.0320	10.00
DU Std. Dev.	0.00	0.00	0.2512	0.000
Diversity Mean	0.0155	0.2055	0.1485	0.0099
Diversity Std. Dev.	0.0904	0.1917	0.1996	0.0860

**Table 5:** Performance statistics for population size = 500.

Metric	No Model	Proposed		
		Contrastive	KL	Contrastive + KL
Nonlinearity Mean	109.739	108.552	108.376	109.5395
Nonlinearity Std. Dev.	0.4937	1.1026	1.0364	0.4778
Diff. Uniformity Mean	10.00	8.0480	8.0720	10.00
DU Std. Dev.	0.00	0.3064	0.3730	0.00
Diversity Mean	0.0198	0.2237	0.2313	0.0119
Diversity Std. Dev.	0.1210	0.2110	0.1802	0.0941

**Table 6:** Performance statistics for population size = 1000.

Metric	No Model	Proposed		
		Contrastive	KL	Contrastive + KL
Nonlinearity Mean	109.9595	109.7665	108.8565	109.5815
Nonlinearity Std. Dev.	0.5273	0.8231	0.6159	0.4066
DU Mean	10.00	10.00	8.6840	10.00
DU Std. Dev.	0.00	0.00	0.9497	0.00
Diversity Mean	0.0182	0.0704	0.3168	0.0165
Diversity Std. Dev.	0.1175	0.2064	0.2405	0.1172

**Table 7:** Summary statistics of S-box optimal performance for population size = 120 and Generation = 1000.

Metric	Mean	Max	Min	Optimized S-Box
Nonlinearity	109.57	110.25	105.25	110.25
DU	9.08	10.00	8.00	8
Diversity	0.175	0.993	0.045	0.993

**Table 8:** Proposed optimized S-box.

226	241	88	252	150	45	163	246	108	176	234	101	216	179	50	98
218	123	27	235	15	146	91	147	75	78	104	69	220	168	116	70
2	144	221	175	199	190	12	155	239	127	213	117	198	204	58	99
205	90	56	162	134	22	245	173	193	143	196	255	174	35	225	24
219	228	10	79	112	8	148	93	33	135	229	210	74	94	51	183
138	211	23	201	5	42	65	238	181	86	29	72	19	36	57	160
142	195	182	191	3	149	137	43	244	44	154	60	95	237	59	120
6	194	207	16	118	242	231	215	192	11	178	212	126	136	180	236
34	54	153	47	243	114	232	89	227	20	167	122	202	68	113	139
159	247	230	62	77	52	200	4	253	251	128	141	100	30	46	110
87	102	164	82	76	7	254	105	129	97	125	172	156	208	131	250
223	184	170	152	140	119	26	186	111	171	224	64	132	18	0	103
66	106	214	73	177	84	206	80	55	1	217	169	158	222	166	48
53	49	197	37	92	187	71	248	161	209	9	28	157	32	107	83
14	203	121	185	130	25	21	240	133	67	31	109	61	81	96	13
124	39	233	145	249	151	165	188	189	17	85	38	115	40	63	41

**Table 9:** Cryptographic performance comparison of optimal S-box with recent state-of-the-art design methods.

Method	Nonlinearity	DU	Diversity
GA [28]	92	10	-
Reinforcement [32]	107.25	12	-
GA [48]	109	10	-
ICO [43]	108	10	-
Booster Algorithm [42]	105.4	10	-
Mutation Optimization [44]	106	10	-
DC-GAN [45]	104	10	-
Multi-Objective Optimization [46]	107.25	10	-
Roulette wheel-based Social Network Search [47]	110	10	-
Proposed (Contrastive + KL) Model	110.25	8	0.993

## Data Availability

The dataset used in this study was generated within the proposed methodology and can be made available upon reasonable request.
